# 0473. Necrosis and apoptosis in liver, spleen, pancreas, kidney and intestinal tissue induced by intra-abdominal hypertension in a porcine model. Second part of an experimental study

**DOI:** 10.1186/2197-425X-2-S1-O15

**Published:** 2014-09-26

**Authors:** JA Buensuseso Alfaro, M Poblano Morales, MA Moreno Eutimio, J Mendoza Escorza, S Zamora Gómez, G Magdaleno Lara, FJ Tendillo Cortijo, M Lomelí Terán, JJ Martínez Mazariegos, E Deloya Tomas, L Torres López, T Mondragon Labelle

**Affiliations:** Hospital Juarez of Mexico, Intensive Care Unit, Mexico, Mexico; Hospital Juarez of Mexico, Learning and Investigation Center, Mexico, Mexico; Sanity Investigation Institute Puerta de Hierro, Medical-Surgery Investigation Unit, Madrid, Spain; Hospital Tec 100, Intensive Care Unit, Santiago de Queretaro, Mexico; Specialty Hospital Better Life ISSTECH, Intensive Care Unit, Tuxtla Gutierrez, Mexico; General Hospital San Juan del Rio, Intensive Care Unit, Santiago de Queretaro, Mexico

## Introduction

Intra-abdominal hypertension (IAH) and abdominal compartment syndrome (ACS) are increasingly recognized as severe complications of critical illness.

## Objectives

Characterize the apoptosis and necrosis in the renal, intestinal, splenic, pancreatic and hepatic tissue in an experimental porcine model after six hours of IAH.

## Methods

Four York-Landrace mixed breed piglets with a mean body weight of 30 kg were obtained from Center for Teaching, Research and Extension in Swine Production, Faculty of Veterinary Medicine, National Autonomous Mexican University.

Biopsies were taken from liver and kidney by laparoscopy prior to induction of IAP, the intra-abdominal pressure was increased with an intra-peritoneal catheter that was placed in the peritoneal cavity at the level of the umbilicus; and then a saline 0.9% solution was infused to increase intra-abdominal pressure. The IAP was increased up to 20 mmHg, renal an liver biopsies were performed by exploratory laparotomy after the increase of IAP to 20 mmHg and 6 hours after sustained elevated IAP.

The biopsy was cut in two pieces and these were immediately placed into a disposable disaggregator Medicon with 50 µm separator mesh plus 1 mL of ice-cold PBS and processed for 50 s in the Medimachine System.

The cell suspension were stained with FitC-conjugated mAb specific for CDXX, PE-conjugated mAb specific for CDXX. Briefly, 1x106 were stained with the fluorochrome-conjugated mAb specific for cell surface antigen markers for 20 min in the dark at 4°C. After incubation, the cells were resuspended in 200 µL PBS for subsequent flow cytometric analysis using a Accuri C6 flow cytometerand then were analysed using FlowJo software V10.

## Results

After 6 hours of sustained IAH there was no difference in the percentage of cells in early apoptosis in the spleen (p=0.19) and intestinal tissue (p=0.24). There was difference in the liver (p=0.01) and pancreas (p=0.02).

**Figure 1:**
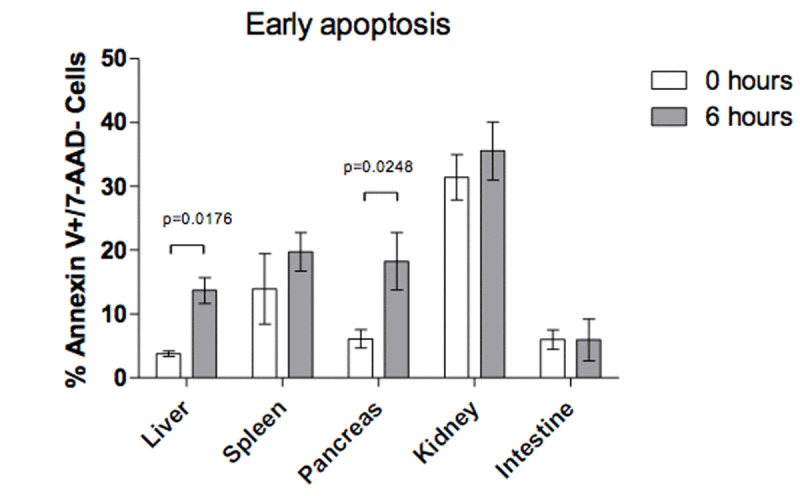
Figure 1

**Figure 2:**
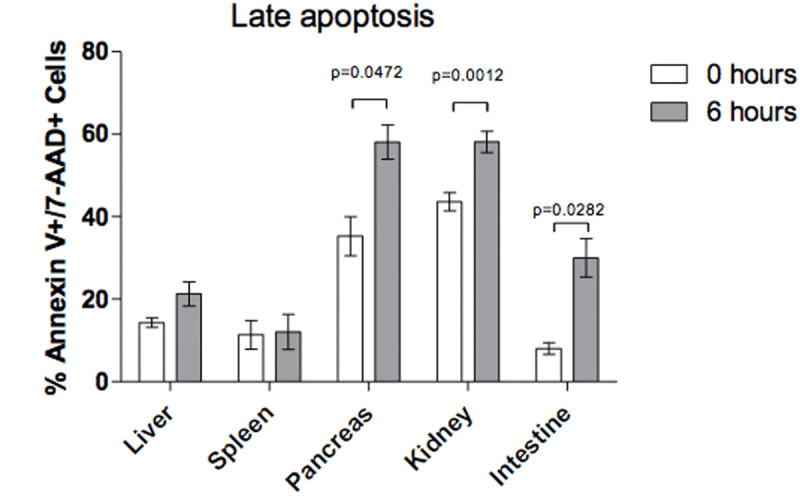
Figure 2

**Figure 3:**
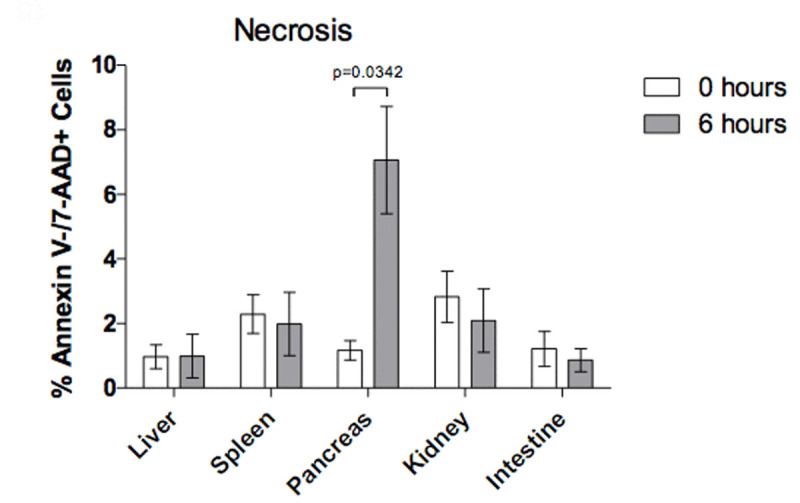
Figure 3

There was difference in pancreas (p=0.04), kidney (p=0.01) and intestinal tissue (p=0.02) after 6 hours of sustained IAH in the percentage of cells in late stage apoptosis, and only the pancreatic tissue shown necrosis (p=0.03) (Fig. 1, Fig. 2, Fig. 3)

## Conclusions

Apoptosis and necrosis presents in pancreas above all, but also in hepatic, splenic, intestinal and kidney tissues after six hours of IAH. Our data suggests that the greatest harm of sustained IAH take place in the pancreas, kidney and intestinal tissue in an early fashion and more lately in the liver.
